# First evidence of subclinical renal tubular injury during sickle-cell crisis

**DOI:** 10.1186/1750-1172-9-67

**Published:** 2014-04-29

**Authors:** Vincent Audard, Stéphane Moutereau, Gaetana Vandemelebrouck, Anoosha Habibi, Mehdi Khellaf, Philippe Grimbert, Yves Levy, Sylvain Loric, Bertrand Renaud, Philippe Lang, Bertrand Godeau, Frédéric Galactéros, Pablo Bartolucci

**Affiliations:** 1 Service de Néphrologie et Transplantation, Institut Francilien de Recherche en Néphrologie et Transplantation (IFRNT), Hôpital Henri-Mondor, Assistance Publique–Hôpitaux de Paris (AP–HP), Université Paris-Est Créteil (UPEC), 51, ave du Marechal-de-Lattre-de-Tassigny, Créteil Cedex, 94010, France; 2 INSERM U955, Equipe 21, Centre de Référence Syndrome Néphrotique Idiopathique, UPEC, Créteil, France; 3 Departement de Biochimie–Pharmacotoxicologie, Hôpital Henri-Mondor, AP–HP, UPEC, Créteil, France; 4 Unité INSERM 955, Equipe 2, UPEC, Créteil, France; 5 Etablissement Français du Sang, Hôpital Henri-Mondor, Créteil, France; 6 Centre de Référence des Syndromes Drépanocytaires Majeurs, Hôpital Henri-Mondor, AP–HP, UPEC, 51, ave du Marechal-de-Lattre-de-Tassigny, Créteil Cedex 94010, France; 7 Service de Médecine Interne, Hôpital Henri-Mondor, AP–HP, UPEC, Créteil, France; 8 Service d’Accueil des Urgences, Hôpital Henri-Mondor, AP–HP, UPEC, Créteil, France; 9 Service d’Immunologie Clinique, Hôpital Henri-Mondor, AP–HP, UPEC, Créteil, France

## Abstract

**Background:**

The pathophysiologic mechanisms classically involved in sickle-cell nephropathy include endothelial dysfunction and vascular occlusion. Arguments demonstrating that ischemia-reperfusion injury-related kidney damage might coincide with vaso-occlusive crisis (VOC) are lacking.

**Methods:**

In this prospective study, we sought to determine whether tubular cells and glomerular permeability might be altered during VOC. Urine neutrophil gelatinase-associated lipocalin (NGAL) levels and albumin-excretion rates (AER) of 25 patients were evaluated prospectively during 25 VOC episodes and compared to their steady state (ST) values.

**Results:**

During VOC, white blood-cell counts (WBC) and C-reactive protein (CRP) were significantly higher than at ST but creatinine levels were comparable. Urine NGAL levels were significantly increased during VOC vs ST (*P* = 0.007) and remained significant when normalized to urine creatinine (*P* = 0.004), while AER did not change significantly. The higher urine NGAL concentration was not associated with subsequent (24-48 hour) acute kidney injury. Univariate analysis identified no significant correlations between urine NGAL levels and laboratory parameters during VOC.

**Conclusions:**

These results demonstrated that subclinical ischemia-reperfusion tubular injury is common during VOC and highlight the importance of hydroelectrolyte monitoring and correction during VOC.

## Background

Sickle-cell nephropathy (SCN), a major mortality risk factor in sickle-cell disease (SCD) patients, is characterized by early increased glomerular filtration rates (GFR), frequently associated with proteinuria, that promote progressive renal function decline [1-3]. Although the SCN pathophysiologic process remains hypothetical, chronic hemolysis-related endothelial dysfunction and the relative renal hypoxia triggered by repeated vaso-occlusive crises (VOC) are currently considered critical key factors [2,4,5]. Experimental data from murine SCD models demonstrated that ischemia-reperfusion (I-R) injury can induce direct kidney damage, characterized by marked renal vascular congestion and tubular injury, associated with renal hemodynamic changes [6,7]. In SCD patients, VOC initiation, progression and resolution may be considered typical features of I-R injury [8,9], but definitive proof of I-R–induced tubular injury in SCD patients during VOC is lacking. Renal tubular cells, a predominant site of cell metabolic activity and oxygen consumption, are highly susceptible to oxygen deprivation during I-R injury [10]. However, clinical demonstration that the kidneys are a major I-R site during SCD VOC remains unsubstantiated. We compared prospective urine neutrophil gelatinase-associated lipocalin (NGAL), a tubular injury biomarker [11], and albumin-excretion rate (AER) levels during VOC to steady state (ST) values to determine whether renal tubular cells and glomerular capillary permeability could be directly affected during VOC.

## Methods

This prospective study, approved by our local Ethics Committee, was conducted in our Adult Sickle-Cell Referral Center in accordance with the Declaration of Helsinki, Good Clinical Practice guidelines, and local laws and regulations. Patients were enrolled after giving their written informed consent.

Homozygous SCD patients, at least 18 years old with severe VOC requiring hospitalization for pain (regardless of location) not controlled by grade-II analgesics, were eligible for inclusion once. VOC was defined as pain or tenderness requiring opioids and not attributable to other causes [12]. Exclusion criteria included: VOC with parenteral hydration lasting >24 hours; blood transfusion during the previous month; acute chest syndrome or severe complication requiring blood transfusion at inclusion; pregnancy and/or psychiatric disorder; preexisting renal insufficiency, defined as GFR ≤60 mL/min/1.73 m^2^ according to the modification of diet in renal disease (MDRD) formula, and/or Hydroxyurea use when the VOC occurred or at ST.

Standardized clinical practices included bed rest, fluid replacement with 5% glucose (2 L with NaCl 4 g/L), oral alkaline water (500 mL/day), folinic acid (5 mg/day), analgesia with morphine and intravenous paracetamol (1 g every 6 hours), and preventive incitative spirometry. Urine NGAL levels and biological parameters were determined simultaneously on samples collected on D1, D2 or D4 of hospitalization for each of the 25 patients’ single VOC and at an ST consultation. ST was defined as a consultation ≥1 months after a VOC and ≥3 months after blood transfusion. The Acute Kidney-Injury Network (AKIN) criteria stratify AKI into 3 stages reflecting severity of the percentage serum-creatinine change [measured using the Jaffe kinetic method (Gen.2, Roche Diagnostics, France)] [13]. All room-temperature urine samples were centrifuged (2000 *g* × 5 minutes) within 2 hours of arriving at the lab and frozen at -80°C until assayed. An enzyme-linked immunosorbent assay kit (BioPorto Diagnostics A/S, Denmark) determined NGAL levels according to the manufacturer’s instructions. AER of <3, 3-30 or >30 mg/mmol creatinine defined normal, micro- or macroalbuminuria, respectively.

### Statistical analyses

Results are expressed as means ± standard deviation or numbers (%). Between-group quantitative parameters were compared with paired Student *t*-tests. Simple linear regressions were computed for each biologic parameter to search for potential relationships with NGAL. Spearman’s correlation coefficient established correlations; *P* < 0.05 defined significance.

## Results and discussion

Twenty-five SS SCD patients (12 men, 13 women; mean age: 34.6 ± 6 years), each hospitalized for a VOC, were included. The mean VOC-onset-to-hospitalization interval was 30.4 ± 23.1 hours. The mean pain visual analog scale at inclusion was 7.2 ± 1.3. None had received nonsteroidal anti-inflammatory drugs (NSAID) to treat VOC. Mean systolic and diastolic blood pressures during VOC were, respectively, 115 ± 13 and 71 ± 9 mm Hg. During VOC, white blood-cell counts (WBC) and C-reactive protein (CRP) were significantly higher than at ST but creatinine levels were comparable (Table 1). During hospitalization, no infection was documented and no patient had AKI satisfying AKIN criteria. The latter observation confirms our reported finding that AKI is rare during VOC [14]. Notably, AKI absence did not exclude possible VOC association with subclinical renal I-R injury.

**Table 1:** Comparisons of biologic parameters during 25 patients’ sickle-cell disease vaso-occlusive crises (VOC) and at steady state (ST)

**Parameter**	**VOC**	**ST**	***P *** **value**
Hemoglobin (g/dL)	8.3 ± 1.3	8.8 ± 1.2	0.01
White blood-cell count (10^6^/L)	12216 ± 4599	9832 ± 3359	0.009
Lactate dehydrogenase (IU/L)	434.8 ± 181.5	392.5 ± 213.1	0.16
Creatinine (μmol/L)	60.3 ± 16.8	57.5 ± 16.1	0.24
Urea (mmol/L)	2.2 ± 1.1	2.9 ± 1.1	0.01
Albuminuria (mg/L)	71.1 ± 259.4	139.4 ± 347.0	0.14
Albuminuria/creatinuria (mg/mmol)	14.6 ± 47.5	18.4 ± 46.9	0.38
Urine NGAL (ng/mL)	76.7 ± 76.7	28.7 ± 25.7	0.007
NGAL/creatinuria (mg/mmol)	17.1 ± 20.7	3.9 ± 3.6	0.004
C-Reactive protein (mg/L)	54.9 ± 60.5	9.1 ± 7.2	0.003

Values are means ± standard deviation.

**Figure 1:**
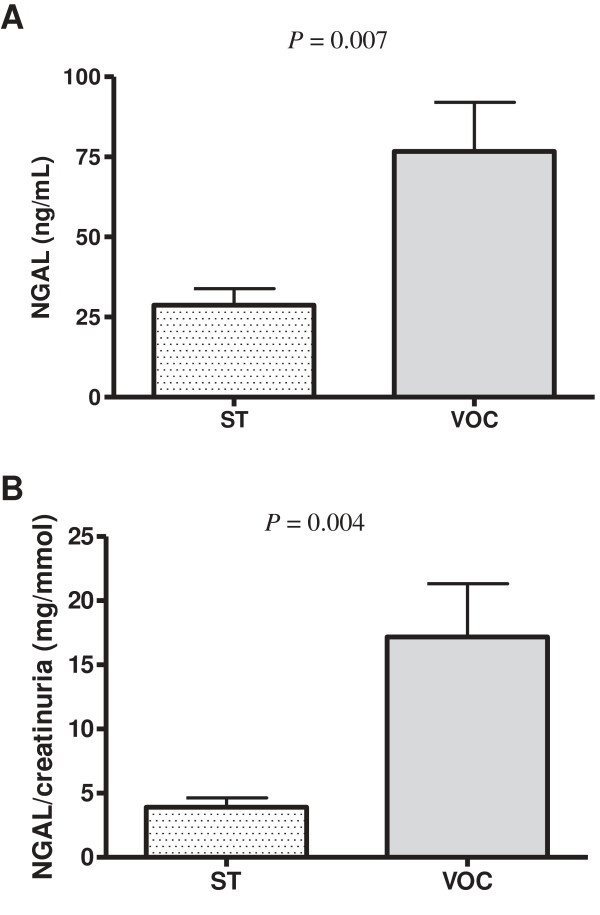
Urine NGAL levels (A) and urine NGAL/creatinine ratios (B) in 25 SCD patients at vaso-occlusive crises (VOC) and steady state (ST).

We next analyzed AER and urine NGAL level changes between VOC and ST. AER, an early marker of renal damage, reflects glomerular endothelial dysfunction and impaired glomerular barrier permeability [15]. It was reportedly elevated in 68% of adult SCD patients [1]. At ST, 15 (60%), 7 (28%) or 3 (12%) patients, respectively, had normal, micro- or macroalbuminuria. VOC and ST AER were comparable (Table 1), suggesting that, during VOC, I-R injury does not directly target glomerular permeability. NGAL, an extracellular, 25-kDa protein, is expressed at low levels in several human tissues, eg kidney [16], and is induced in tubular cells early after renal ischemic injury [17]. Urine or serum NGAL levels are considered relevant biomarkers of AKI [16,18]. Patients with preexisting renal insufficiency were excluded from our analysis because recent findings suggested that urine and serum NGAL concentrations increased in parallel with renal disease severity [19]. Moreover, NSAID, known to cause tubular injury, were not used because our previous prospective, controlled trial results showed limited ketoprofen impact on VOC requiring hospitalization [12]. Sundaram et al recently demonstrated that ST urine NGAL levels were subnormal in 116 SCD patients and independent of the degree of albuminuria [20]. Herein, urine NGAL levels increased significantly during VOC (*P* = 0.007) and remained significant when normalized to urine creatinine (*P* = 0.004) (Figure 1). NGAL concentrations on hospital D1, D2 or D4 (n = 9, 11 and 5, respectively) were comparable (*P* = 0.9). Median urine NGAL values were significantly associated with AKI stage in a cohort of patients without AKI or with stage-3 AKIN admitted to the emergency department (respectively, 23 vs 153 ng/mL) [21]. Our patients’ mean NGAL-level increase in urine samples collected a mean of 30.4 ± 23.1 hours after VOC onset was relatively weak (76.7 ng/mL), probably reflecting subclinical renal injury, because none had AKI meeting AKIN criteria [22].

Based on the 50 samples from 25 patients, no correlation was found between urine NGAL levels and other laboratory parameters considered (WBC counts, hemoglobin, CRP, lactate dehydrogenase, ASAT, bilirubinemia and creatinine levels). These findings suggest that acute tubular injury during VOC is not directly related to hemolysis, anemia and inflammatory syndrome that may confound NGAL level interpretation in these patients [16]. Furthermore, they highlight the importance of correcting hydroelectrolyte disturbances, particularly acidosis, which raises the hemoglobin desaturation rate and favors HbS polymerization. Indeed, our therapeutic protocol for VOC includes bicarbonate administration. The first objective of our study was not to determine whether a potential increase of urine NGAL levels might be predictive of subsequent acute kidney injury (AKI) in these patients, as we previously demonstrated that AKI during VOC is a rare finding [14]. Our study’s primary aim was to determine whether increased urine NGAL levels might represent a pertinent biomarker attesting to subclinical tubular impairment in these patients. In agreement with that hypothesis, as observed herein, Haase et al recently identified, among 2322 critically ill patients, 19.2% failing to meet current creatinine-based consensus criteria for AKI but probably having acute tubular injury, based on increased NGAL levels [22]. We postulate that repeated tubular injury in the context of multiple VOC might promote hypoxic stress, thereby inducing a chronic inflammatory state in renal parenchyma, leading to progressive tubulointerstitial and glomerular damage [4,23].

## Conclusions

This study is the first to indicate that I-R tubular damage might occur simultaneously with VOC, as revealed by increased urine NGAL levels. The demonstration that tubular cells are indeed a major site of tissue-oxygen deprivation during SCD VOC is important for VOC management and emphasizes the need to improve intravenous hydration and patients’ tissue oxygenation during VOC. Further investigations are needed to evaluate a potential direct relationship between elevated NGAL levels during VOC and the risk of subsequent chronic kidney disease.

## Competing interests

The authors declare no competing interests.

## Author’s contributions

Contributions: PB and VA designed the study, analyzed and interpreted data, wrote the manuscript; SM, GV and SL conducted laboratory tests and reviewed data; AH, MK, PG, YL and BR, contributed patients and collected data; PL, PG, BG and FG reviewed the data and the paper; all authors approved the final manuscript.
